# L-Carnitine as a Diet Supplement in Patients With Type II Diabetes

**DOI:** 10.7759/cureus.7982

**Published:** 2020-05-05

**Authors:** Dimitrios T Karalis, Tilemachos Karalis, Stergios Karalis, Angeliki S Kleisiari

**Affiliations:** 1 Nutrition and Dietetics, University of Thessaly, Volos, GRC; 2 Obstetrics and Gynecology, General Hospital of Trikala, Trikala, GRC; 3 Internal Medicine, General Hospital of Trikala, Trikala, GRC; 4 Nutrition and Dietetics, University of Thessaly, Trikala, GRC

**Keywords:** carnitine, types 2 diabetes, dietary supplements, nutritional intervention, diabetes management

## Abstract

Introduction: L-Carnitine is a very important component of the human body which is involved in cardiac function and generally in the proper functioning of the muscular system. Also, it contributes to the proper use of glucose by the cell, thereby improving the regulation of glucose metabolism of the diabetic patient and preventing complications such as fatigue, insomnia, and mental activity. In this paper we would like to show the therapeutic effect of L-carnitine on type II diabetic patients after 2 g/day oral administration of L-carnitine.

Methods: In this study 181 Greek patients, 84 men and 97 women, aged 50-65 years, Type II diabetics, were administered L-carnitine for six months. All of them were euglycemic, under the proposed treatment, with no diabetic complications or cardiovascular problems. They were under the Mediterranean diet trying to keep their body mass index (BMI) constant. They were neither smokers nor alcohol drinkers. They were administered 2 g/day L-carnitine, orally, once daily for six months, on an empty stomach. The blood tests included fasting glucose, glycated hemoglobin (HBA1c), total cholesterol, and triglycerides and they were performed before, three months after, and six months after the treatment initiation. We also evaluated their tiredness, insomnia, and mental activity at these time points; the participants were given forms to fill out (regarding the distance they are able to brisk walk thrice/week, the duration of their calm uninterrupted sleep and their performance in a cognitive screening test, respectively) and based on the results of their answers, they were allocated to graded groups and scale analysis was performed in each one of them.

Results: Fasting glucose mean decrease was 17.51 after three months of medication (p<0.05); the decrease though noted after six months was not statistically significant. HbA1c showed a statistically significant mean decrease in both three- and six-month milestones (0.335, p<0.05 and 0.623, p<0.05 respectively). Changes noted in cholesterol levels were not statistically significant. Triglyceride measurements showed a significant decrease; -15.38 after three months (p<0.05) and -31.39 after six months of treatment (p<0.05). Finally, significant changes were found in both time periods for tiredness (three months: -0.49, p<0.05, six months: -0.88, p<0.05), insomnia (three months: -0.49, p<0.05, six months: -0.88, p<0.05), and mental activity (three months: +0.25, p<0.05, six months: +0.89, p<0.05).

Conclusion: L-Carnitine could be a valuable dietary supplement in patients with type II diabetes who follow a Mediterranean diet and are under recommended treatment. Research in this field though is at an early stage and more studies should be performed.

## Introduction

Carnitine is a water-soluble carbonic acid that is exclusively synthesized in the liver and kidney from the basic amino acids lysine and methionine [1]. Its name comes from the Latin word carnus which means flesh. It was discovered in 1905 in meat extracts by the Russian scientists Gulewitsch and Krimberg and by the German researcher Kutscher [2]. In biological systems it occurs in two forms: as nonesterified (free) carnitine and as esterified (acylcarnitine) carnitine [3]. In the mid-20th century, carnitine was considered as a vitamin because it was found to be a necessary growth factor for a flour moth, known as Tenebrio Molitor, and given the name vitBt [4]. In humans it is a vital element, but it cannot be considered as a vitamin even if its involvement in cardiac function is very important [5-6].

Carnitine is absorbed through the intestinal mucosa. According to Kendler, niacin, pyridoxine, ascorbic acid, and divalent iron are essential for its biosynthesis [7]. That is, the lack of these elements by the human body can lead to carnitine deficiency. Main sources of nutrition are meat, poultry, and dairy products; other sources of lower content are fish and breast milk and even lower extent plant-based foods [8]. More than 95% of the body's total carnitine storage exists within skeletal muscle tissue [9]. An increase in levels of skeletal muscle’s carnitine after intake with carbohydrates was found [10].

After intravenous administration of L-carnitine to healthy euglycemic volunteers, an increase of 17% in glucose use was detected with 50% increase in unoxidized glucose disposal [11]. Other studies have shown an increase in insulin sensitivity and glucose disposal in obese patients following administration of L-carnitine and at the same time an improvement in the development and management of cardiovascular complications on type II diabetes [12-13].

In 1973 carnitine deficiency was recognized as the cause of myopathy [14]. Also, the effect of carnitine on the rate of neural transmission of the posterior tibial nerves, central ulnar, and gastrocnemius has been demonstrated [15]. Lack of carnitine in the body reduces pain sensation and reduces energy and endurance by transferring fatty acids to mitochondria for ATP production [10, 16]. It is also considered to be effective in the treatment of depression, possibly due to its involvement in lipid metabolism, which is an important factor in the pathophysiology of depression [17].

Carnitine is known to improve glycemic regulation by increasing peripheral glucose utilization [18]. Its use is not only involved in regulating the lipidemic profile but also in improving the function of the myocardium and skeletal muscle [19-20]. There is also evidence for significant difference between physical fitness and resistance to fatigue after carnitine use and improved mental activity [21].

All the above contributed to the formulation of this study, in order to evaluate the effects of carnitine administration on type II diabetics and its impact on glycemic and lipidemic profile of these patients, and at the same time to measure possible changes in muscle and mental fatigue as well as on mental activity.

## Materials and methods

The study involved 181 Greek patients, 84 men and 97 women, aged 50-65 years. All were type II diabetics, relatively euglycemic, under treatment according to the proposed protocols, with no diabetic complications and no cardiovascular problems. They were under nutrition monitoring (Mediterranean diet) trying to stay consistent in their eating habits and body mass index (BMI). The study excluded smokers and alcohol drinkers. All of the participants were questioned prior to the study and reported an unstressful way of living.

All patients were administered L-carnitine orally, at a dosage of 2 g/day, once daily on an empty stomach. The patients received the treatment for six months. The results were measured and evaluated three times; at the beginning of the study, after three months of L-carnitine treatment, and after six months of L-carnitine treatment. The values measured are the following: fasting glucose, glycated hemoglobin (HbA1C), total cholesterol, triglycerides, tiredness, insomnia, and mental activity.

Colorimetric method was used to determine fasting glucose (TARGA BT3000 biochemical analyzer was used, reported normal values: 75-115 mg/dL). HbA1c was determined by the high-performance liquid chromatography (HPLC) method (desirable percentage <7%). Blood total cholesterol and triglycerides were measured by the enzymic colorimetry method (analyzer reported normal values: 150-200 mg/dL for cholesterol, 20-170 mg/dL for triglycerides). The evaluation of insomnia, tiredness, and mental activity was based on group formulation and scale analysis on data derived from the participants after filling the corresponding form. In order to evaluate sleep, three groups were formulated (<5 h/day, 5-6 h/day, >7 h/day) and patients were allocated to their corresponding group after reporting the duration of their continuous uninterrupted night sleep. In order to evaluate tiredness, all patients were asked to follow an exercise schedule of uninclined brisk walking three times preweek; three groups were formulated (2-3 km, 3-4 km, >4 km) and patients were allocated to their corresponding group after reporting at the time of the evaluation the number of kilometers they are able to brisk walk. Finally, mental activity was assessed using Montreal Cognitive Assessment (MoCA) test, and based on the patient's score on each evaluation, they were allocated in one of the three groups that were formed (group A: 26-27, group B: 28-29, group C: 30).

Participants were fully informed and agreed to the study through written consent (according to the protocols of the National Ethics Committee for Clinical Studies).

## Results

The results were studied using the Statistical Package for the Social Sciences software (IBM SPSS Statistics, Version 25). The study included 181 participants (sample size N=181).

**Figure 1:**
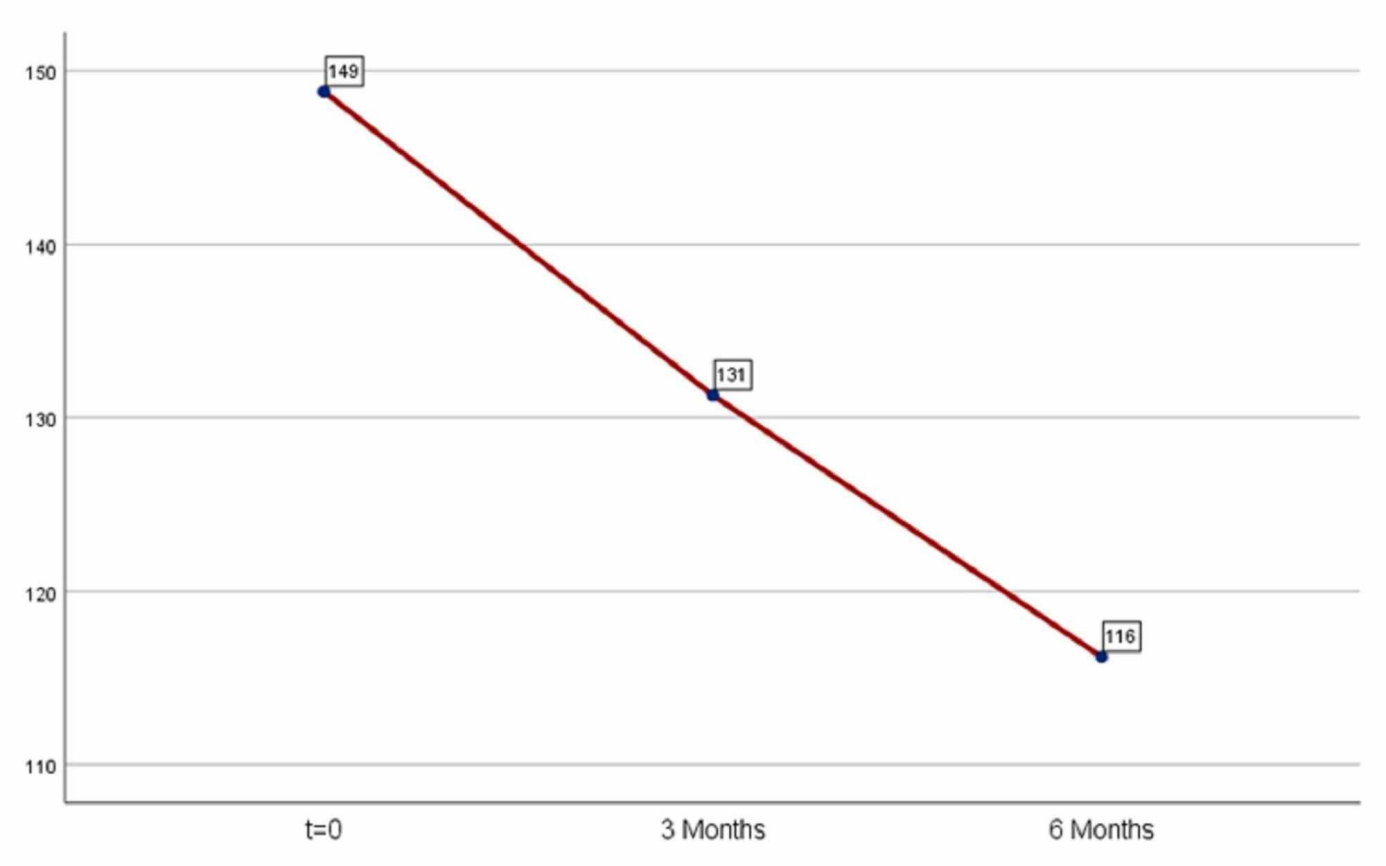
Average glucose fasting levels.

The administration of medication after three months led to a significant decrease in fasting glucose: 17.51 (mean decrease=17.51). The results were statistically significant (p=0.00001<0.05). After six months of administration, the decrease of fasting glucose was 32.59 (mean decrease=32.59) compared to the day 0; however, six months decrease was not statistically significant as the index p exceeds 0.05 (p=0.067). Results are shown in Table 1 and Figure 1.

**Table 1:** Glucose fasting descriptive statistics (zero, three, and six months).

	N	Minimum	Maximum	Mean	Std. deviation
Glucose fasting	181	110	234	148.80	22.476
Glucose fasting after three months	181	87	184	131.29	18.329
Glucose fasting after six months	181	84	168	116.21	12.852
Valid N (listwise)	181				

**Figure 2:**
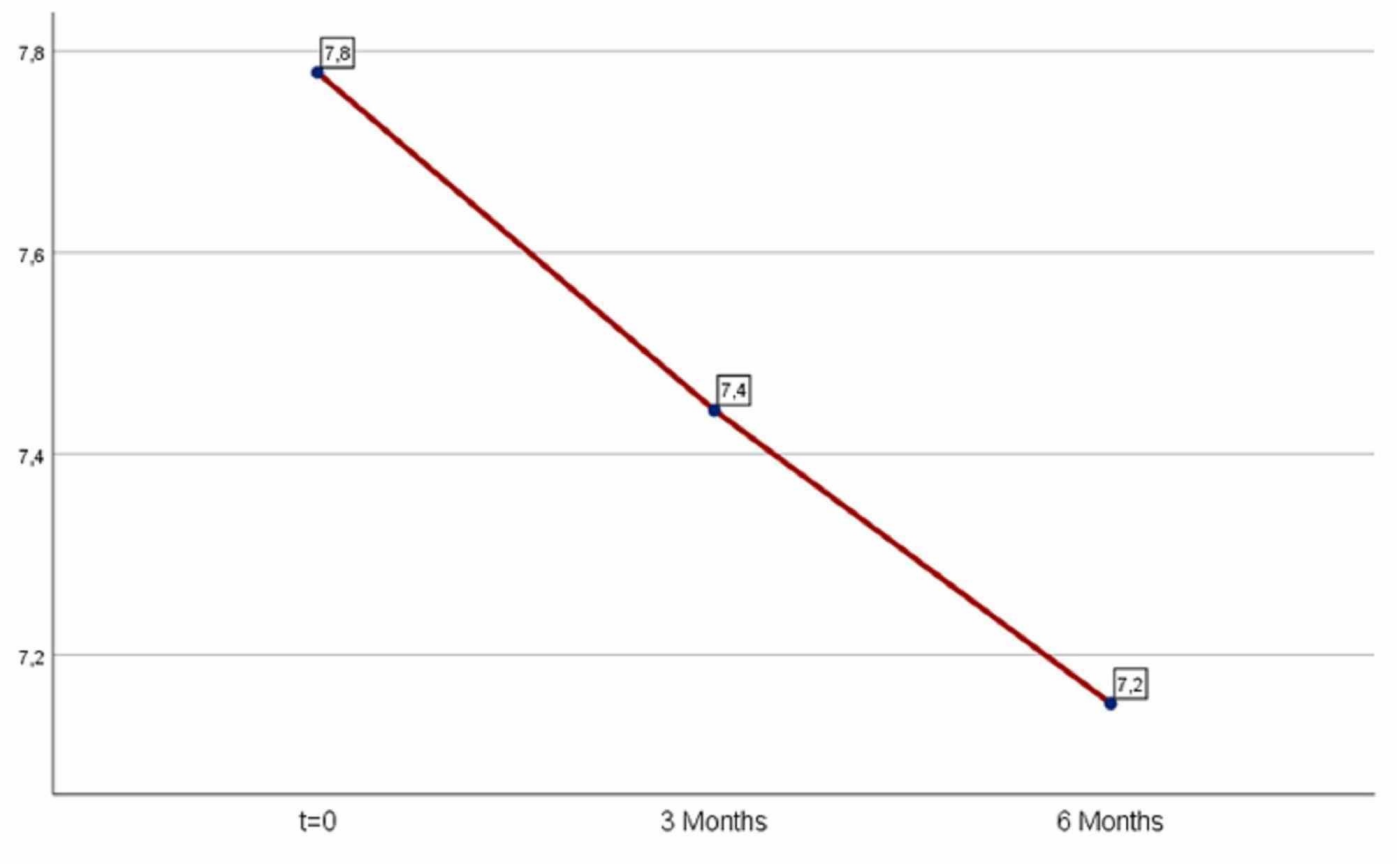
Average HbA1c levels. HbA1c, glycated hemoglobin

The decrease in HbA1c levels was significant: 0.335 (mean=0.335) after the first trimester and 0.623 (mean=0.623) after six months as shown in Table 2 and Figure 2. The results were statistically significant in both periods (first period: p=0.00001<0.05, second period: p=0.00001<0.05).

**Table 2:** HbA1c descriptive statistics (zero, three, and six months). HbA1c, glycated hemoglobin

	N	Minimum	Maximum	Mean	Std. deviation
HbA1c	181	5.6	10.1	7.775	0.7723
HbA1c after three months	181	5.0	9.7	7.440	0.6818
HbA1c after six months	181	5.5	8.6	7.152	0.5618
Valid N (listwise)	181				

**Figure 3:**
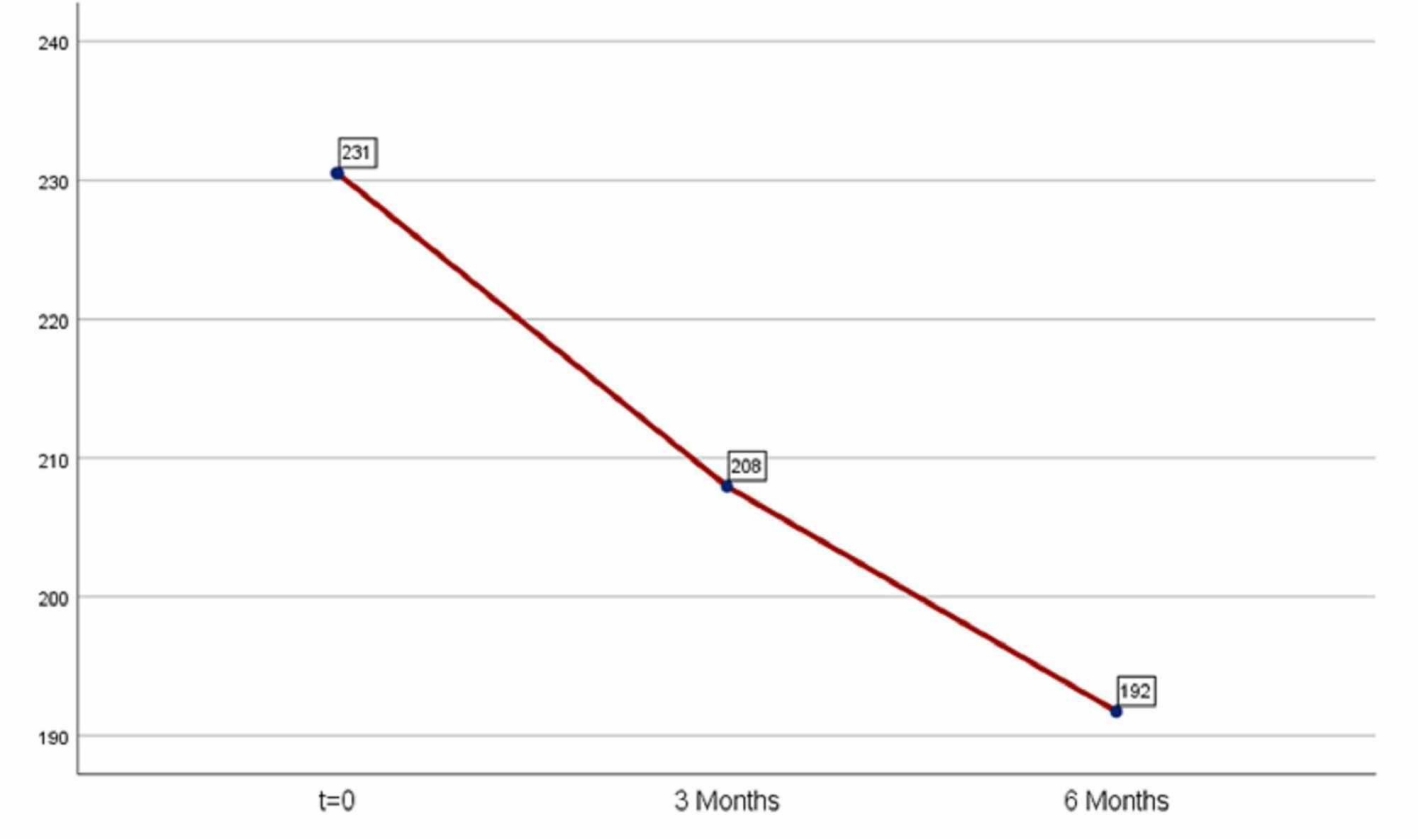
Average cholesterol levels.

The levels of total cholesterol according to mean value decreased. In the first three months cholesterol levels were decreased by 22.5 (mean=22.5) and in the six months by 38.77 (mean=38.77). However, the results are not statistically significant because the index p exceeds 0.05 in both periods (first period: p-value =0.08>0.05, second period: p-value=0.387>0.05). As a result, medication is not considered effective for this index as shown in Table 3 and Figure 3.

**Table 3:** Cholesterol descriptive statistics (zero, three, and six months).

	N	Minimum	Maximum	Mean	Std. deviation
Cholesterol	181	178	307	230.51	32.125
Cholesterol after three months	181	147	268	207.96	25.648
Cholesterol after six months	181	148	250	191,.4	17.903
Valid N (listwise)	181				

**Figure 4:**
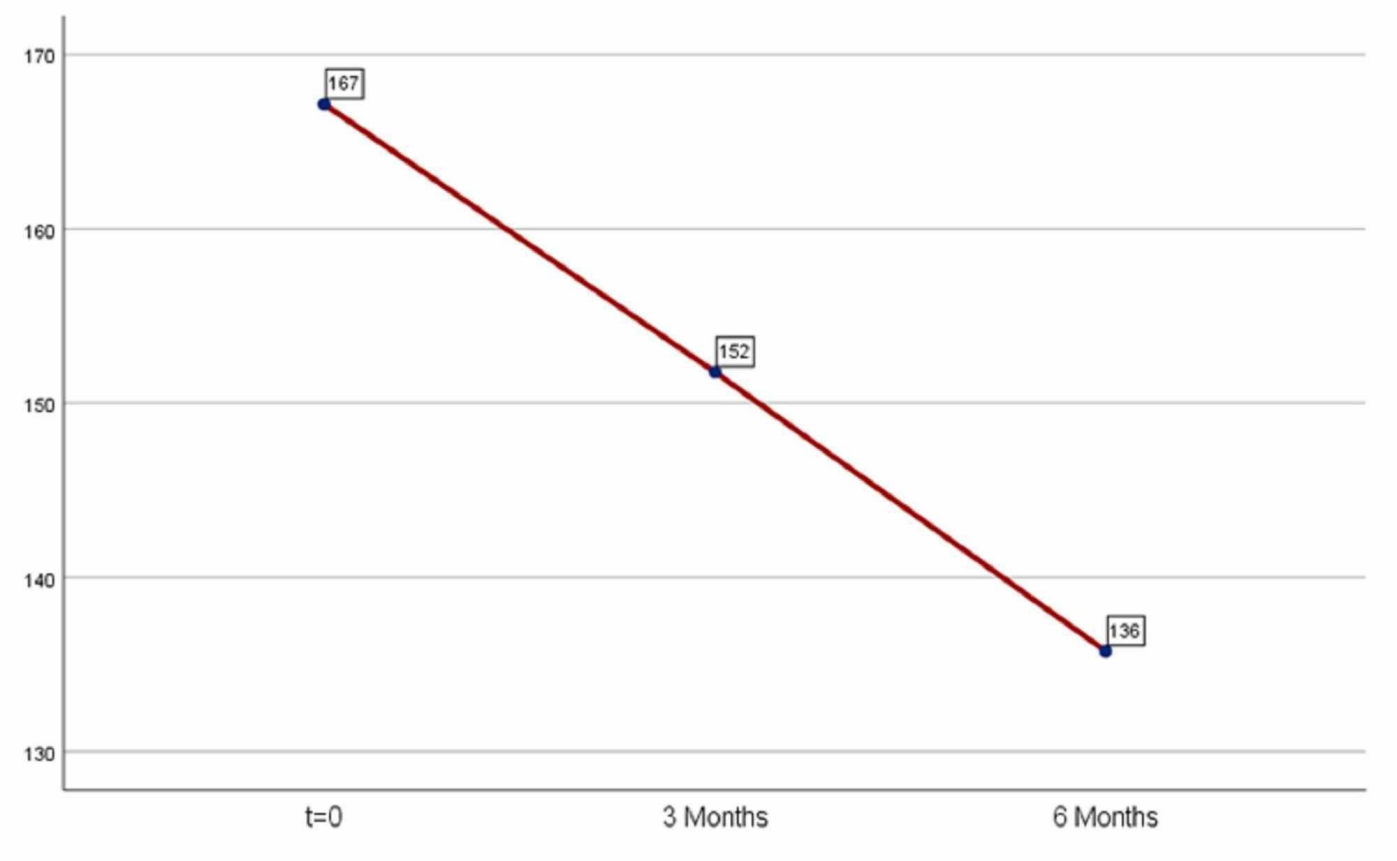
Average triglyceride levels.

For triglyceride measurements the results were statistically significant in both periods. The administration of medication after three months led to a significant decrease in triglycerides: 15.38 (mean=15.38) and continued to drop after six months of medication (mean=31.39) as shown in Table 4 and Figure 4. The results were statistically significant in both periods (first period: p=0.00001<0.05, second period: p=0.00001<0.05).

**Table 4:** Triglycerides descriptive statistics (zero, three, and six months).

	N	Minimum	Maximum	Mean	Std. deviation
Triglycerides	181	82	227	167.16	26.707
Triglycerides after three months	181	75	260	151.78	26.083
Triglycerides after six months	181	71	225	135.77	24.974
Valid N (listwise)	181				

**Figure 5:**
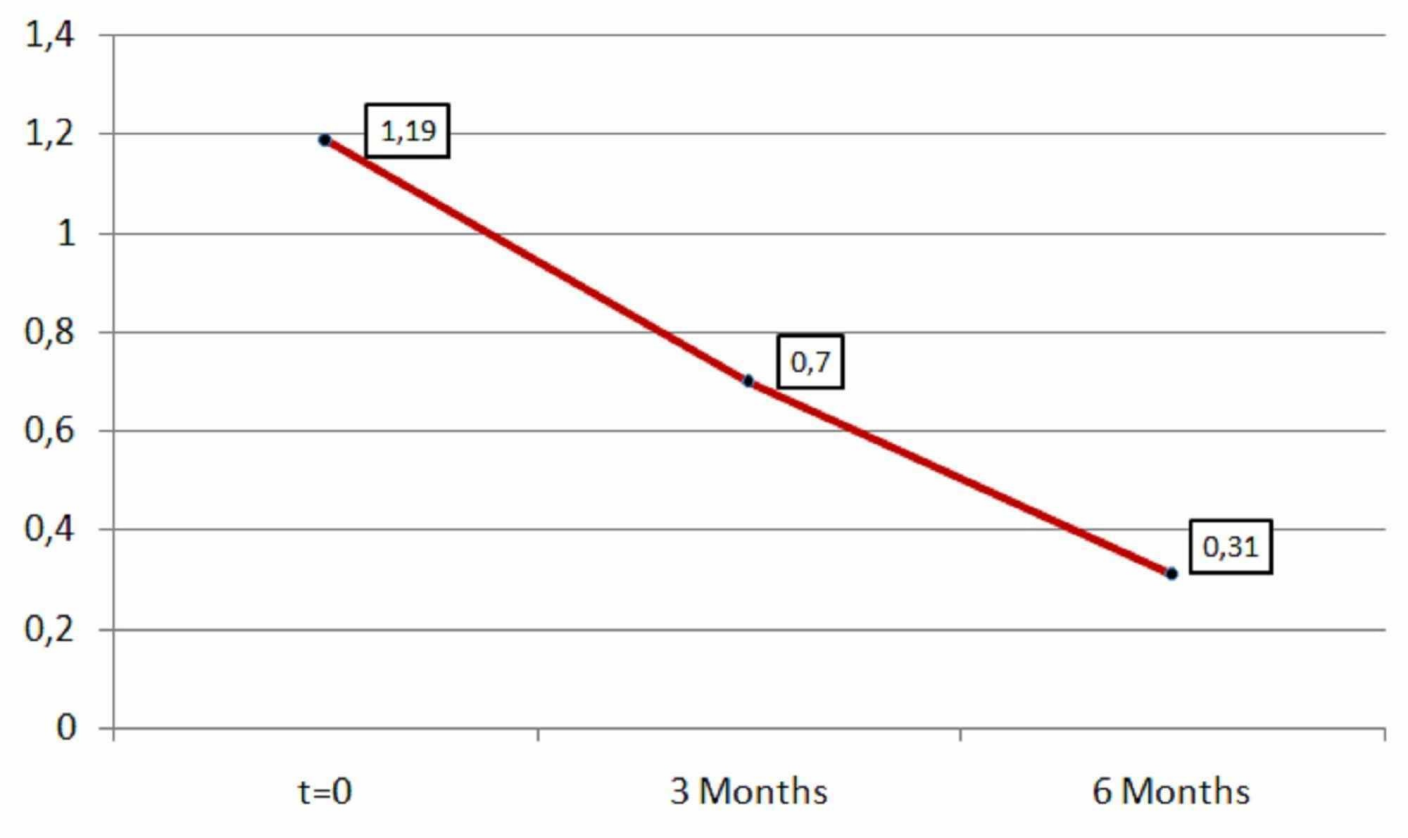
Average tiredness.

Moreover, the average value of tiredness decreased by 0.49 (mean=0.49) after three months of medication with L-carnitine and continued to drop after six months of medication (mean=0.88). The results were statistically significant in both periods (first period: p=0.00001<0.05, second period: p=0.00001<0.05). Therefore, the difference in tiredness as observed in the two time periods is highly significant as shown in Table 5 and Figure 5.

**Table 5:** Tiredness descriptive statistics (zero, three, and six months).

	N	Minimum	Maximum	Mean	Std. deviation
Tiredness	181	0	2	1.9	0.556
Tiredness after three months	181	0	2	0.70	0.659
Tiredness after six months	181	0	2	0.31	0.475
Valid N (listwise)	181				

**Figure 6:**
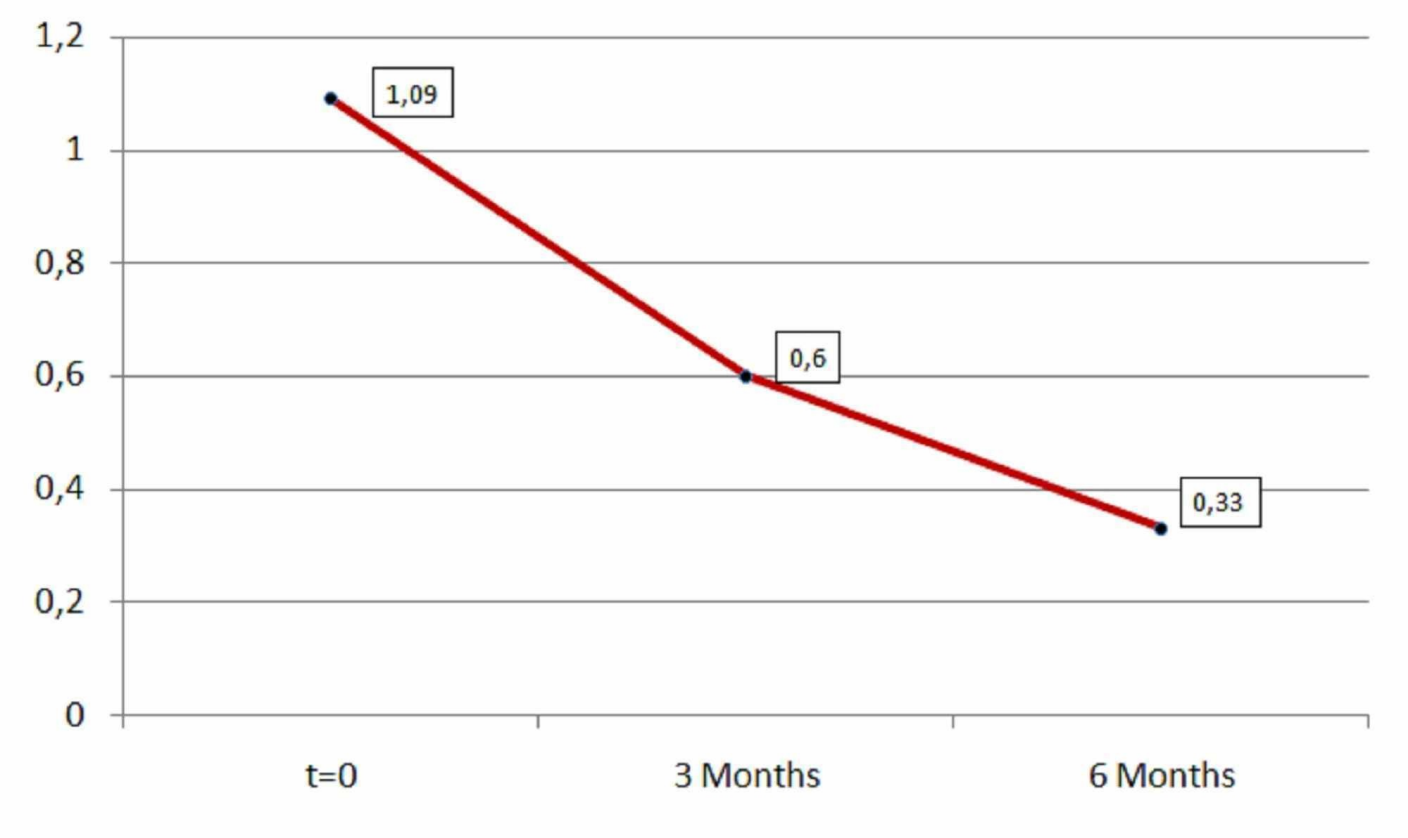
Average insomnia.

According to research, L-carnitine has also a positive effect on insomnia. After three months of L-carnitine, the decrease of insomnia was 0.49 (mean=0.49) and after six months was 0.88 (mean=0.88) as shown in Table 6 and Figure 6. The results were statistically significant in both periods (first period: p=0.00001<0.05, second period: p=0.00001<0.05).

**Table 6:** Insomnia descriptive statistics (zero, three, and six months).

	N	Minimum	Maximum	Mean	Std. deviation
Insomnia	181	0	2	1.09	0.565
Insomnia after three months	181	0	2	0.60	0.585
Insomnia after six months	181	0	2	0.33	0.482
Valid N (listwise)	181				

**Figure 7:**
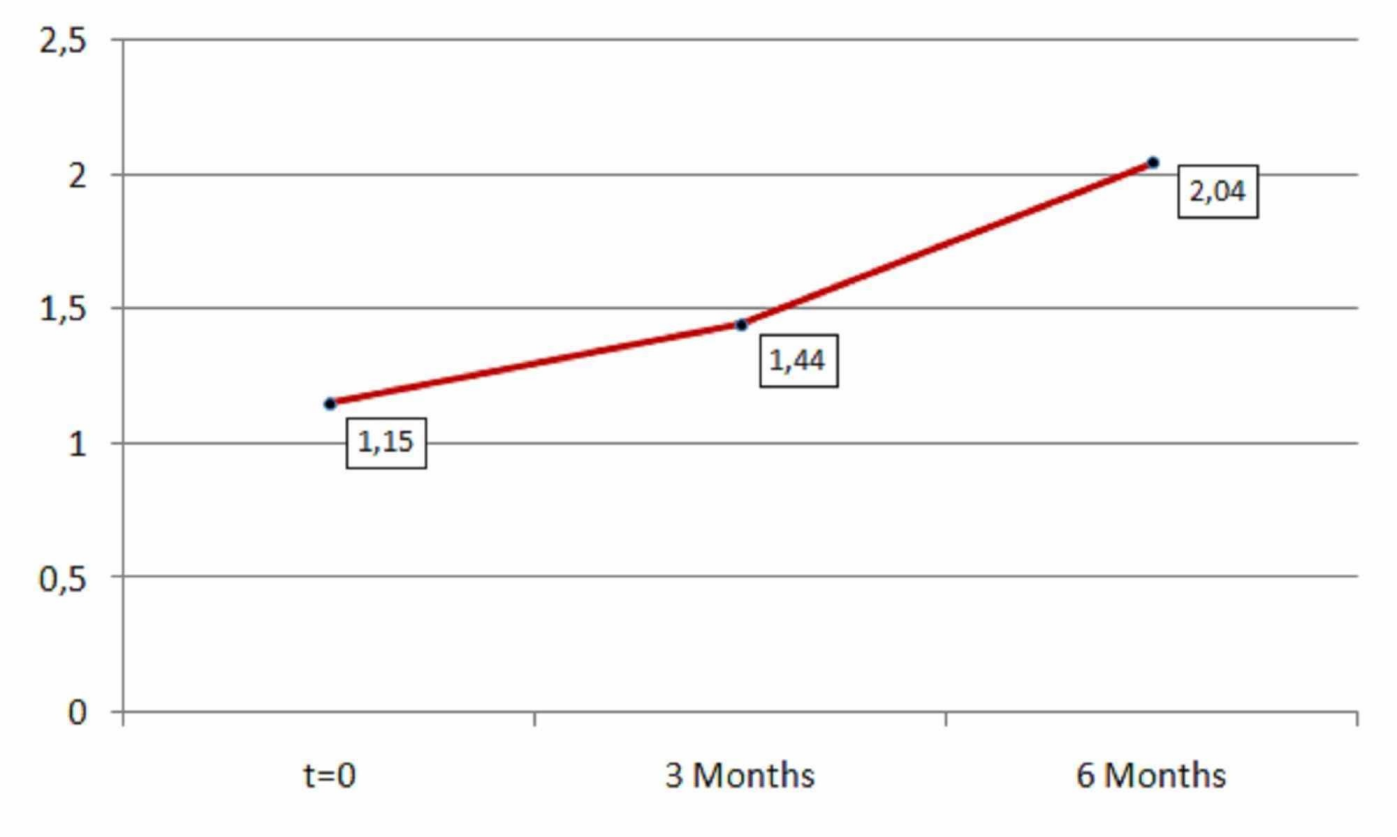
Average mental activity.

Finally, the mental activity increased significantly (first period: p=0.00001<0.05, second period: p=0.03<0.05) both for the time period of three months (mean=0.25) and for the time period of six months (mean=0.89) as shown in Table 7 and Figure 7.

**Table 7:** Mental activity descriptive statistics (zero, three, and six months).

	N	Minimum	Maximum	Mean	Std. deviation
Mental activity	181	1	2	1.15	0.363
Mental activity after three months	181	1	3	1.44	0.571
Mental activity after six months	181	1	3	2.04	0.670
Valid N (listwise)	181				

## Discussion

Our study has shown that administration of L-carnitine has a series of effects to both lipid and glycemic profile of type II diabetics, as well as to their physical exercise capability, sleep quality, and mental activity. Analysis of the results has demonstrated statistically significant changes on both three months and six months after the initiation of the treatment in HbA1c levels (decrease), triglyceride levels (decrease), tiredness estimation (decrease), insomnia estimation (decrease), and mental activity estimation (increase). Fasting glucose levels showed a statistically significant decrease three months after L-carnitine administration, but the following decrease on six months appeared to have no statistical significance. Finally, cholesterol levels changes were not statistically significant on any time in our experiment.

The results have potential clinical interest, as they suggest that a simple addition to the medication of type II diabetes mellitus patients of a nutrition supplement could actually alter many parameters of the disease primary and secondary effects, and benefit the patients in both their biochemical profile and in terms of life quality.

## Conclusions

The present study attempted to demonstrate the possible benefits of carnitine administration as a dietary supplement in patients with type II diabetes, under the condition of following the Mediterranean diet as a recommended treatment option, and managed to detect a series of variable changes in the glycemic and lipid profile of these patients. However, research in this field is at an early stage and more studies should be made to support our findings.
